# A Rare Case of Clear Cell Adenocarcinoma of the Cervix with No Intrauterine Diethylstilbestrol Exposure

**DOI:** 10.7759/cureus.7796

**Published:** 2020-04-23

**Authors:** Vinay Mathew Thomas, Swetha Ann Alexander, Matthew J Hadfield, James Vredenburgh

**Affiliations:** 1 Internal Medicine, University of Connecticut Health Center, Farmington, USA; 2 Internal Medicine, University of Connecticut, Farmington, USA; 3 Hematology and Oncology, Saint Francis Hospital and Medical Center, Hartford, USA

**Keywords:** cervical clear cell carcinoma, clear cell adenocarcinoma of the cervix, clear cell cancer, cervical cancer, diethylstilbestrol, cisplatin, paclitaxel

## Abstract

Cervical cancer is the fourth most common cancer in females. Clear cell adenocarcinoma of the cervix is an uncommon histological variant and is usually seen with intrauterine exposure to diethylstilbestrol. A 28-year-old female with no intrauterine exposure to diethylstilbestrol presented with postcoital bleeding. A pelvic exam revealed a cervical mass. Imaging confirmed the cervical mass and positron emission tomography scan showed an increased uptake in the cervical mass as well as the para-aortic and pelvic lymph nodes. Biopsy showed a clear cell carcinoma of the cervix. She was treated with cisplatin and paclitaxel for eight cycles and concurrent radiation therapy. She had a complete response to therapy and has been in complete remission nine months from the end of therapy. There are no clear guidelines for the treatment of clear cell carcinoma with current therapy based on the treatment of squamous and non-clear cell adenocarcinoma. Cisplatin and paclitaxel could be an option, given the successful treatment of the patient in our case.

## Introduction

Cervical cancer poses a significant toll on the global cancer scene, being the fourth most common cancer in females. Cervical tumors arising from the ectocervix are most commonly squamous cell carcinomas and those arising from the endocervix are commonly adenocarcinomas. Clear cell carcinoma is a less common histological variant [1]. Clear cell adenocarcinoma of the cervix (CCAC) has classically been associated with intrauterine exposure to diethylstilbestrol (DES) [2]. However, there have been reported cases of clear cell carcinoma of the cervix without any identifiable exposure to DES. The etiology and pathogenesis associated with CCAC remain unclear. The presentation is variable, with vaginal bleeding being a common presentation [3]. Since it presents in young females, it can sometimes be misdiagnosed as functional vaginal bleeding [4]. This can often result in a delay in diagnosis. Because of the rarity of the condition, there are no established guidelines for the treatment. Current treatment methods are derived from the experience of treatment with squamous cell and non-clear cell adenocarcinomas. Depending on the stage of the disease, fertility-preserving treatment can also be tried [5].

We present a patient with CCAC who presented with postcoital bleeding and successfully completed treatment with weekly cisplatin and paclitaxel in combination with radiation therapy.

## Case presentation

**Figure 1:**
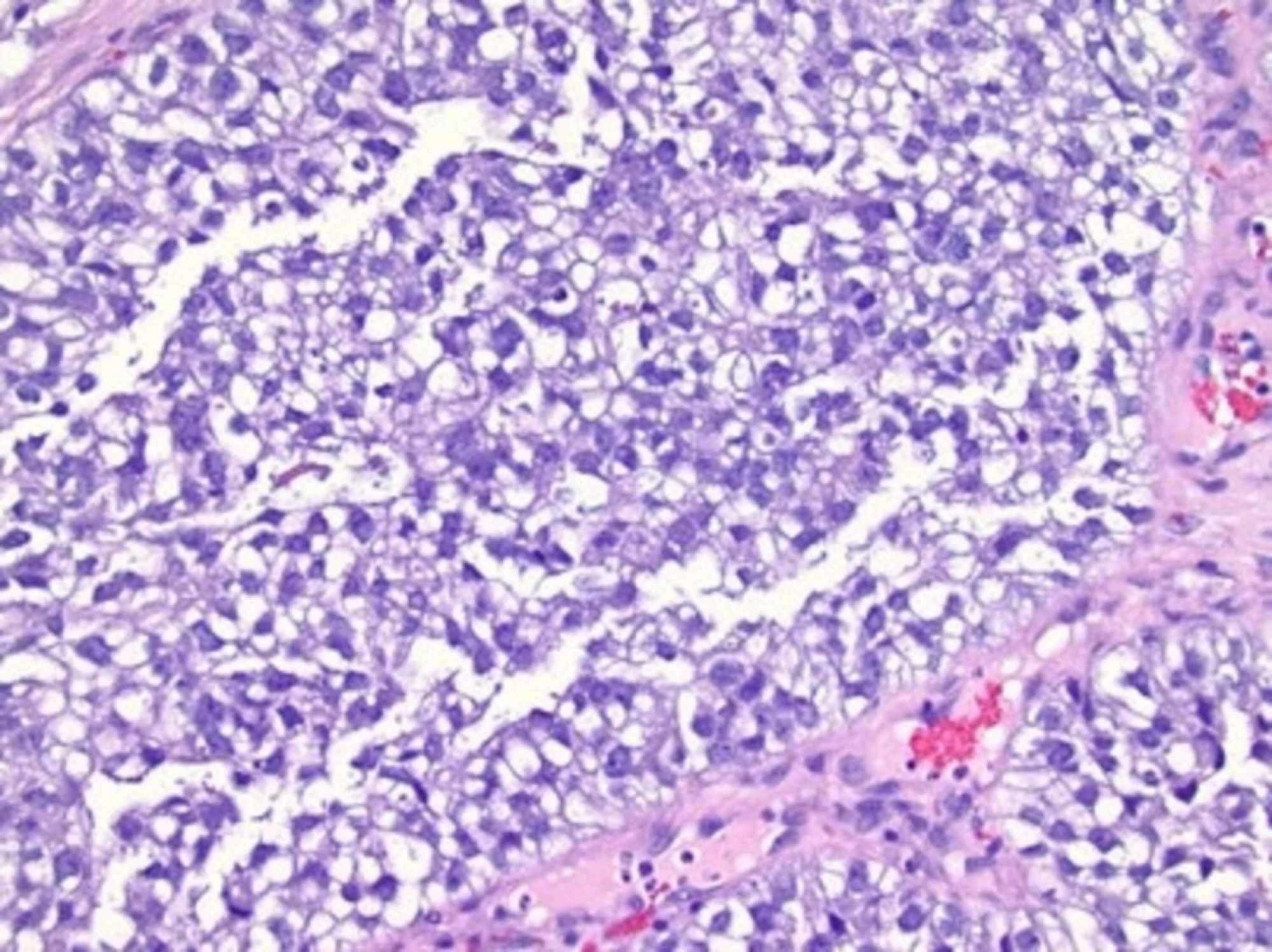
Biopsy of the cervical mass showing large neoplastic cells with ovoid nuclei and clear cytoplasm consistent with clear cell carcinoma

A 28-year-old female with no significant past medical history presented to her gynecologist with postcoital bleeding. A pap smear was performed that revealed a normal-appearing cervix. Over the next several months, the patient began having vaginal bleeding more frequently, occurring almost daily. A pelvic exam performed at that time revealed a cervical mass, around 6 cm. A pap smear was performed, and there was abnormal histology showing atypical glandular cells, suspicious for malignancy. HPV (human papillomavirus) testing was negative. A uterine ultrasound was ordered, which showed the uterus measuring 3.67 x 5.54 x 4.88 cm, endometrium 3.41 mm, cervix 3.04 cm, right ovary 1.6 x 3.66 x 1.94 cm, and left ovary 1.58 x 3.16 x 1.69 cm. Echogenic fluid was noted in the cervical region with no free fluid identified. A biopsy of the mass showed large neoplastic cells with ovoid nuclei and clear cytoplasm, consistent with clear cell carcinoma (Figure 1).

**Figure 2:**
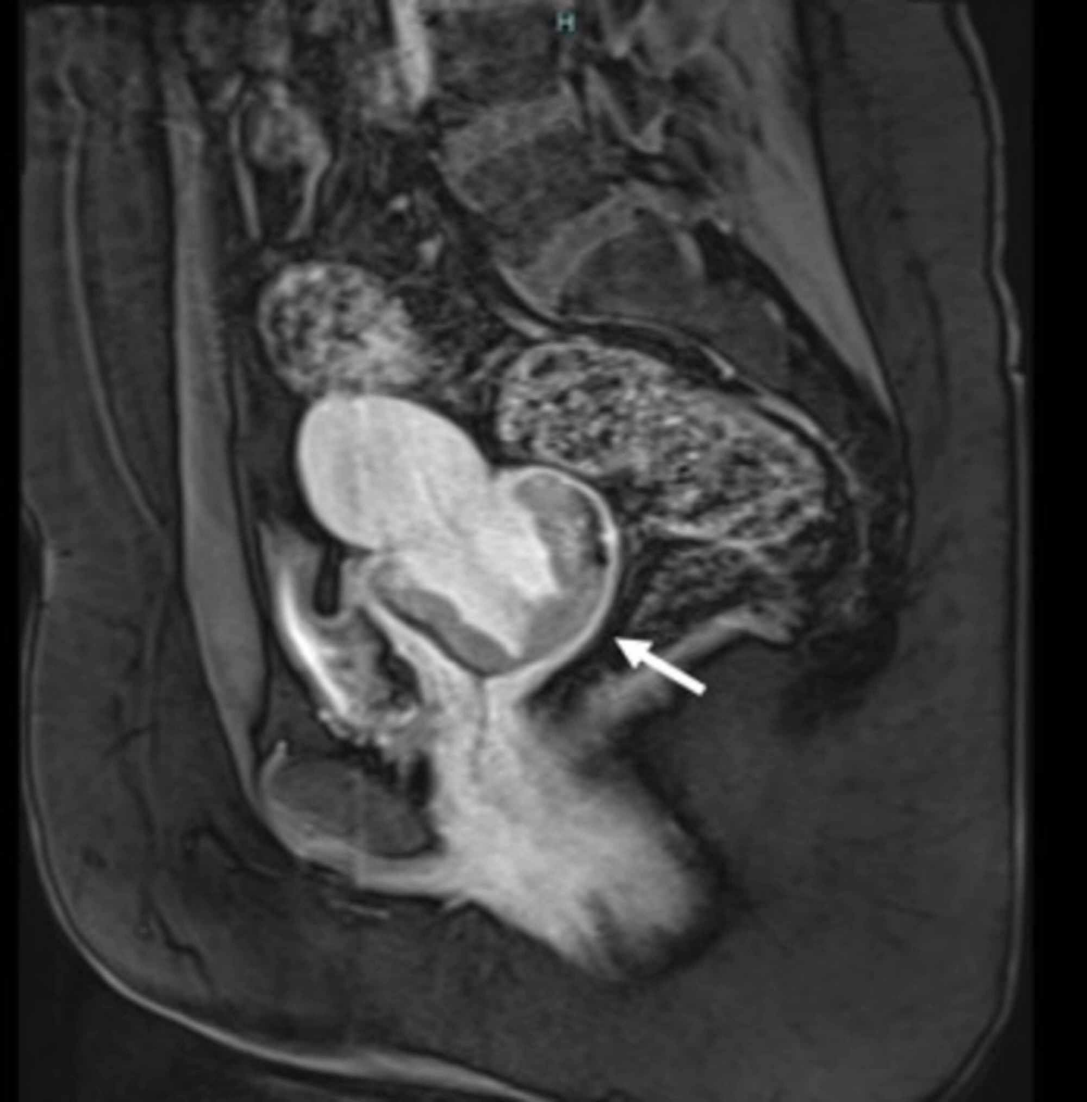
Pelvic MRI before treatment, showing the cervical mass projecting into the vagina

Immunomarkers were negative for p16, Vimentin, CD10, CDX2, CK20, Napsin A, and EFP. Periodic acid-Schiff was strongly positive in the cytoplasm consistent with glycogen, which again pointed toward clear cell carcinoma. The patient’s mother did not have a history of DES exposure in utero. The patient was born several years after the FDA ban on DES use in pregnancy, which made this history reliable. The patient denied risk factors such as multiple sex partners, HPV infection in the past, and smoking. Pelvic MRI was performed to further delineate the mass. The MRI showed a cervical mass measuring 6.5 x 5.6 x 4 cm projecting in the vagina with no parametrial invasion (Figure 2). 

**Figure 3:**
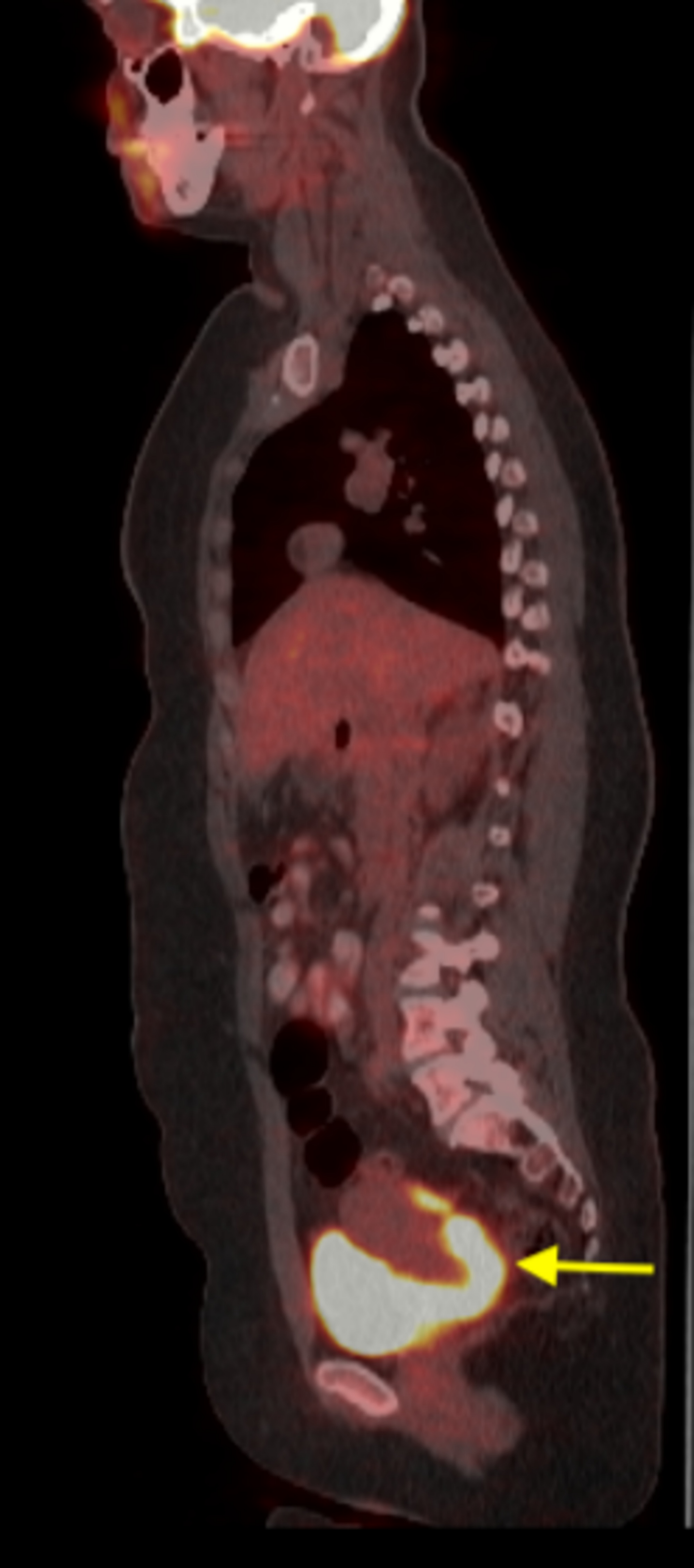
Positron emission tomography scan showed increased metabolic activity in cells on the cervical surface

The upper uterine segment and ovaries appeared normal on MRI and a 1.0-cm left external iliac lymph node was appreciated. The patient underwent a metastatic workup including positron emission tomography (PET) imaging. PET imaging showed increased metabolic activity in cells on the cervical surface, corresponding to the cervical cancer as well as in the para-aortic and pelvic lymph nodes (Figure 3).

**Figure 4:**
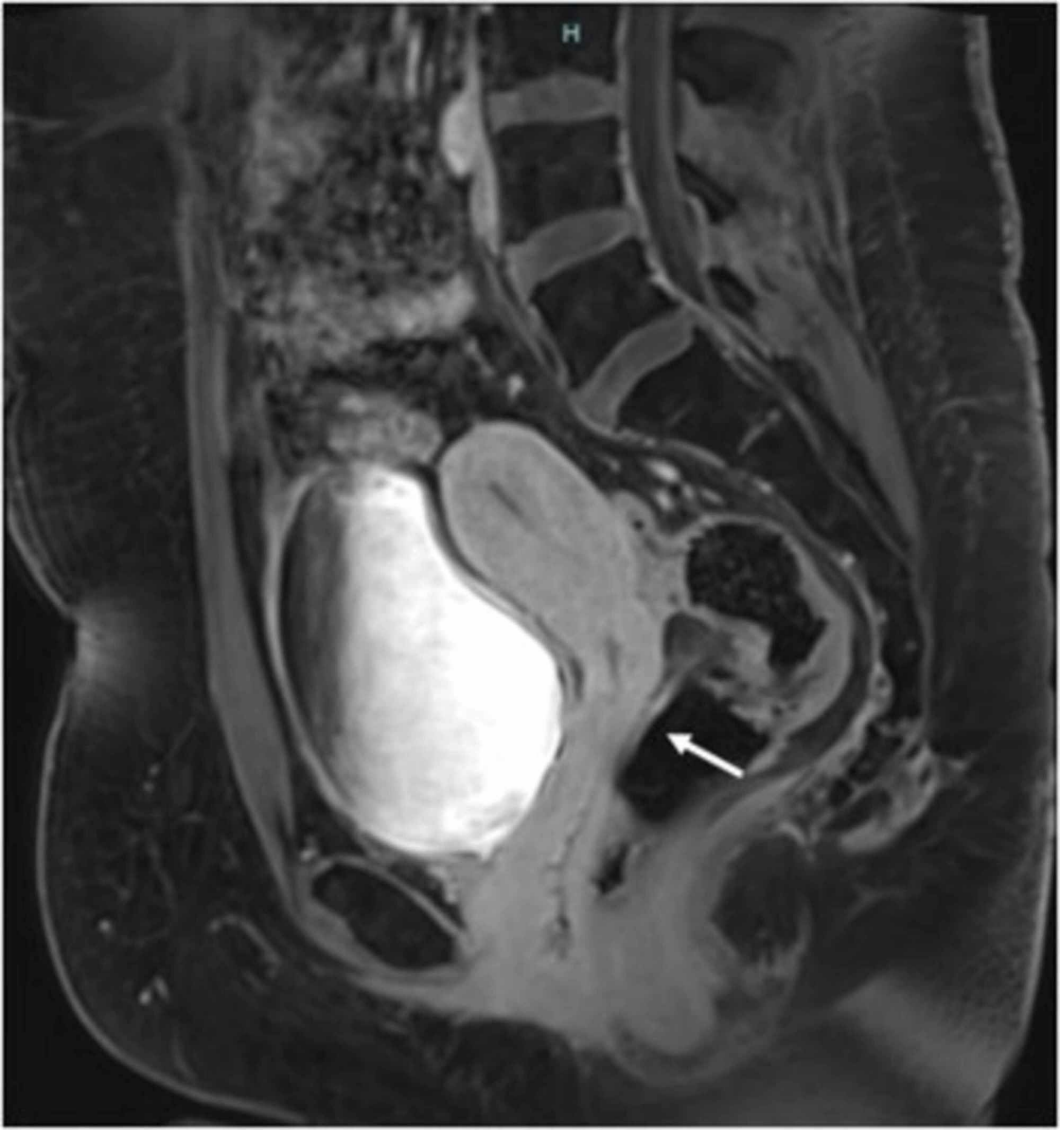
Pelvic MRI after treatment showing resolution of the cervical mass

Also, there was an increased uptake in the bilateral ovaries, which raised the concern of ovarian metastasis versus a primary ovarian malignancy versus functional uptake. The patient underwent a bilateral salpingo-oophorectomy with omental/peritoneal biopsies and diaphragm smears. The subsequent pathology reports revealed no ovarian carcinoma. There was no evidence of malignancy in the omental/peritoneal biopsies and also the diaphragm smears. The patient was diagnosed with FIGO stage IB2 (T1b2N1M0) clear cell carcinoma of the cervix. She was treated with cisplatin at a dose of 30 mg/m^2^ and paclitaxel at a dose of 50 mg/m^2^ for a total of eight cycles. She was also given concurrent external beam radiation therapy to the pelvic and para-aortic lymph nodes and also intracavitary brachytherapy. She received a total dose of 5,580 centigray (cGY) of radiation. She then underwent an MRI to assess treatment and she was found to have resolution of the cervical mass and also the para-aortic and pelvic lymph nodes (Figure 4). 

**Figure 5:**
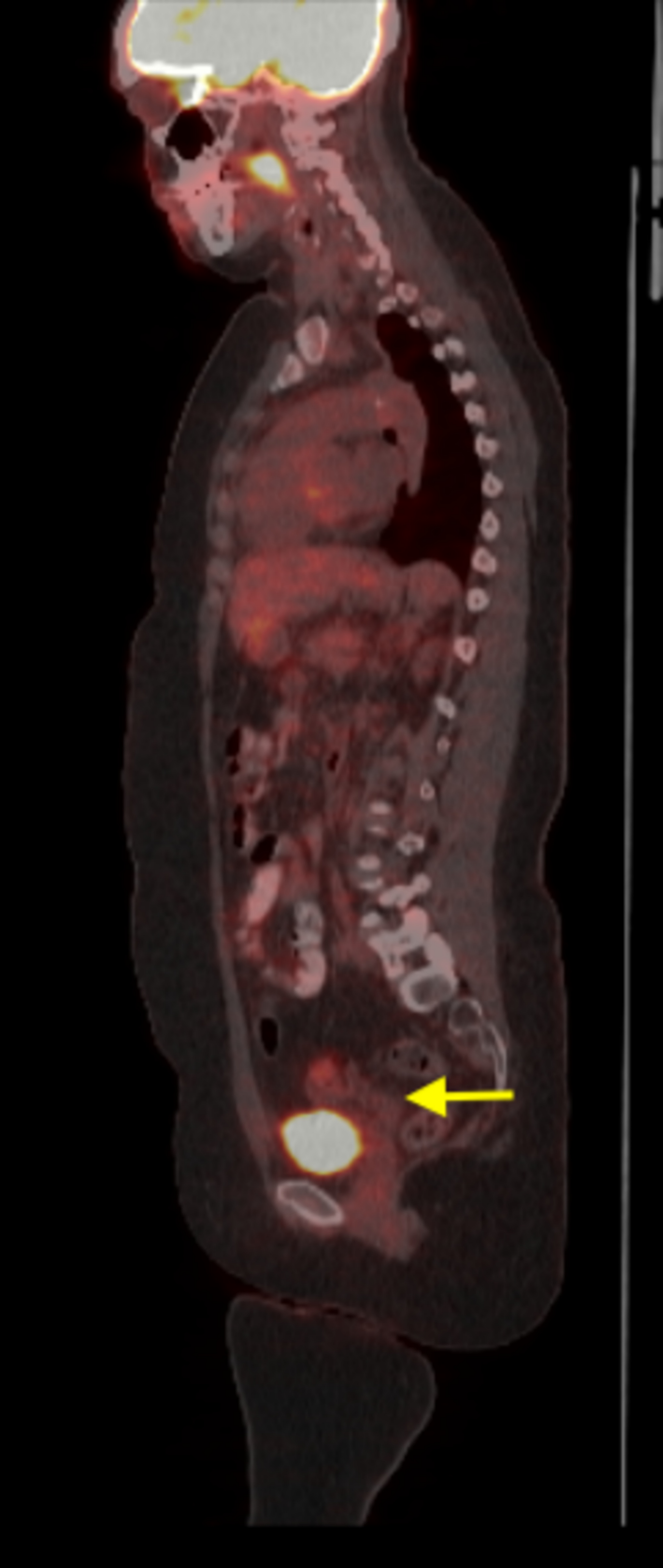
PET scan after treatment showing no evidence of local or distant metastatic disease

The patient also underwent a PET scan after the treatment, and there was no evidence of local or distant metastatic disease (Figure 5). The patient continues to follow with oncology and has had a complete response to treatment and is currently nine months from the end of the combined therapy with no recurrence noted. 

## Discussion

Cervical cancer is the fourth most common cause of female cancers worldwide, both in terms of incidence and death toll [1]. There is significant geographical variability in the incidence and deaths caused by cervical cancer. Incidence is higher in areas with a lower Human Development Index, such as South East Asia and Sub-Saharan Africa [1].

With regard to the anatomy of the cervix, the transitional layer between the squamous and columnar epithelium, called the squamocolumnar junction, is believed to be at the greatest risk of malignant transformation. Squamous cell carcinomas of the cervix are more common than adenocarcinoma of the cervix, with the squamous cell variant accounting for 75% of invasive cervical cancers [1]. Clear cell carcinomas account for 4%-9% of adenocarcinomas of the cervix [6].

One of the major risk factors associated with the development of CCAC was intrauterine DES exposure. DES was a synthetic estrogen that was used by pregnant women to prevent miscarriages, premature labor, and other pregnancy-related complications. In 1971, Herbst and colleagues demonstrated a clear association between the development of vaginal clear cell carcinoma and intrauterine DES exposure [2]. This led the FDA to ban the use of DES in pregnancy (in 1971). This further led to the establishment of the US Registry for Research on Hormonal Transplacental Carcinogenesis. Herbst, in 1999, reviewed 705 cases of clear cell adenocarcinoma which included both vaginal and cervical clear cell adenocarcinomas. Of these cases, 60% of cases had a clear exposure to intrauterine DES, 30% did not have DES exposure, and 10% had an unclear exposure history [7]. The etiology and risk factors for the development of non-DES related CCAC remain unclear. The risk factors associated with squamous cell carcinoma such as multiple sexual partners, HPV infection, increased age, and smoking are not associated with CCAC [8,9]. Pirog et al conducted a global study in which samples were collected from all over the world in order to determine the prevalence of HPV infection in different histological subtypes of cervical adenocarcinomas. Of the 682 cases of adenocarcinomas that were found to be eligible for the study, there were around 30 cases (4.4%) of clear cell carcinoma. Of these 30 cases, six cases were found to be HPV positive [10]. Waggoner and colleagues in 1994 conducted a study to see if there was an association between HPV positivity and CCAC and vagina. However, there was no clear association that could be made [11]. It is unclear at this point whether HPV infection is a cofactor in the development of CCAC or just a coincidental finding. Further studies are required to find the association between CCAC and HPV infection. Cervical endometriosis has also been postulated as a risk factor for the development of CCAC since endometriosis is a risk factor for ovarian CCAC. There have been reports of CCAC arising in cervical endometriosis; however, no clear association is present [12]. p53 mutations and microsatellite repeats causing genomic instability have also been implicated in CCAC [13].

Hanselaar and colleagues analyzed the data registry in the Netherlands and noted that there was a twin peak incidence of CCAC at ages 26 and 71 years [14]. It is important to note that this included both DES-exposed and DES non-exposed patients. The early peak corresponded to patients who had been exposed to DES [14]. In a study done by Thomas and colleagues, which included 94% DES non-exposed patients, the mean peak incidence age was found to be 53 years [9]. This suggests that CCAC is more common in elderly post-menopausal women. Vaginal bleeding is the most common presentation of CCAC [9]. In pediatric patients, there can often be a delay in the diagnosis of CCAC. This could be because physicians feel hesitant performing per-vaginal exams in young girls and adolescents, and also because there is a tendency to diagnose young girls with vaginal bleeding with functional uterine bleeding [15]. It has been demonstrated that CCAC mostly shows an endophytic rather than an exophytic growth pattern, which can result in a normal pelvic exam [6]. This also explains the reason of a low rate of abnormal pap smears in CCAC, with some studies showing rates as low as 18% [6,9]. CCAC shows different microscopic patterns, which include solid, tubulocystic, papillary, or a combination of features. In the solid pattern, the cells have cytoplasm which is clear and glycogen-rich with atypical nuclei. In the tubulocystic pattern, there are tubules and cystic spaces lined by clear cells. The papillary pattern is the least common type. The best outcome is associated with the tubulocystic pattern and the worst outcome with the solid pattern [8]. In a study by Reich and colleagues, it was identified that CCAC spread to the uterine corpus more commonly than other forms of cervical cancer [6]. This is important because spread to the uterine corpus portrays a negative prognosis. In the same study, it was noted that CCAC did not have an increased rate of spread to the vagina when compared to other types of cervical cancer. Common sites of lymphatic spread included the parametrial (40%) and pelvic lymph nodes (47%) [6]. Metastasis to the pelvic nodes increases the risk of involvement of para-aortic lymph nodes. In the study by Thomas and colleagues, it was noted that all patients with positive para-aortic lymph nodes had pelvic lymph node involvement [9]. Thus, it is suggested that para-aortic lymph node dissection be done in patients with pelvic lymph node involvement in CCAC [9]. Studies have suggested that ovarian metastasis is more common in adenocarcinoma of the cervix, but this is not applicable to CCAC. Studies have failed to show an increased risk of ovarian metastasis in CCAC [6]. Lung, liver, and bone are the extrapelvic sites of metastasis that have been identified [6].

The treatment of CCAC is not well defined and is largely based on the methods used to treat squamous cell carcinoma and non-clear cell adenocarcinoma. In the early stages, surgery is an option. There has been an increasing focus on fertility-preserving treatment. Vaginal radical trachelectomy and abdominal radical trachelectomy have emerged as viable options in early stages (IA-IB1) of cervical cancer without lymphatic spread, in women below 45 years of age and with a strong desire to preserve fertility [16]. In more advanced stages, surgery is not recommended, since it is unlikely that it will be curative. Also, advanced stages require adjuvant chemotherapy and radiotherapy, and if patients have had surgery, it is associated with a higher risk of complications [17]. In our patient as well, we did not proceed with surgery because of the advanced stage and was instead treated with chemotherapy and radiotherapy. The chemotherapy of choice is usually weekly cisplatin along with radiation. Combination chemotherapy with cisplatin and paclitaxel could also be an option, as in our patient, who had a complete response to the cisplatin and paclitaxel plus radiation. One study also suggested that there was an increased activation of the EGFR-PI3K-AKT-mTOR pathway in CCAC and that inhibitors of tyrosine kinase and AKT-mTOR may be novel therapeutic targets [18].

CCAC by itself does not indicate a poor prognosis. Reich and colleagues did not note any statistical difference in the five-year survival of CCAC when compared to squamous cell carcinoma of the cervix and non-CCAC [6]. The five-year survival rate for all stages of CCAC ranges from 40% to 72.2% [19]. Poor prognostic factors include advanced-stage CCAC, with Thomas and colleagues reporting three-year survival rates as high as 91% in early-stage cervical cancer (stage I-stage IIA) compared to 22% in stage III or IV [9]. Hanselaar and colleagues noted that reported tumor size > 4 cm was an important negative prognostic indicator [14]. Nodal involvement has a negative impact on the overall survival (31% vs 92%) and progression-free survival (80% vs 100%) [10]. Nuclear atypia, increased mitotic activity, and a solid growth pattern all indicate a poor prognosis [9]. The median recurrence is around 12 months [10]. However, CCAC is known for late recurrences, with some studies reporting recurrences more than eight years after treatment [7]. This necessitates prolonged follow-up of patients with CCAC.

## Conclusions

CCAC without DES exposure continues to be a rare disease. With its similarity of presentation to anovulatory bleeding in young women, and often presenting without a cervical mass, it requires an astute physician to clinch the diagnosis. With no clear guidelines for the management of CCAC, there needs to more randomized control trials for finding the appropriate treatment regimen. But the rarity of the condition might prove to be a challenge in conducting large-scale trials.
